# Effectiveness of an image analyzing AI-based Digital Health Technology to identify Non-Melanoma Skin Cancer and other skin lesions: results of the DERM-003 study

**DOI:** 10.3389/fmed.2023.1288521

**Published:** 2023-10-06

**Authors:** Helen Marsden, Caroline Morgan, Stephanie Austin, Claudia DeGiovanni, Marcello Venzi, Polychronis Kemos, Jack Greenhalgh, Dan Mullarkey, Ioulios Palamaras

**Affiliations:** ^1^Skin Analytics Ltd., London, United Kingdom; ^2^Dermatology Unit, University Hospitals Dorset, Poole Hospital, Poole, United Kingdom; ^3^Dermatology Unit, University Hospitals Sussex NHS Foundation Trust, Brighton, United Kingdom; ^4^Department of Dermatology, Barnet and Chase Farm Hospitals, Royal Free London NHS Foundation Trust, London, United Kingdom

**Keywords:** skin cancer, Artificial Intelligence, Digital Health Technology, skin lesions, smartphone cameras

## Abstract

**Introduction:**

Identification of skin cancer by an Artificial Intelligence (AI)-based Digital Health Technology could help improve the triage and management of suspicious skin lesions.

**Methods:**

The DERM-003 study (NCT04116983) was a prospective, multi-center, single-arm, masked study that aimed to demonstrate the effectiveness of an AI as a Medical Device (AIaMD) to identify Squamous Cell Carcinoma (SCC), Basal Cell Carcinoma (BCC), pre-malignant and benign lesions from dermoscopic images of suspicious skin lesions. Suspicious skin lesions that were suitable for photography were photographed with 3 smartphone cameras (iPhone 6S, iPhone 11, Samsung 10) with a DL1 dermoscopic lens attachment. Dermatologists provided clinical diagnoses and histopathology results were obtained for biopsied lesions. Each image was assessed by the AIaMD and the output compared to the ground truth diagnosis.

**Results:**

572 patients (49.5% female, mean age 68.5 years, 96.9% Fitzpatrick skin types I-III) were recruited from 4 UK NHS Trusts, providing images of 611 suspicious lesions. 395 (64.6%) lesions were biopsied; 47 (11%) were diagnosed as SCC and 184 (44%) as BCC. The AIaMD AUROC on images taken by iPhone 6S was 0.88 (95% CI: 0.83–0.93) for SCC and 0.87 (95% CI: 0.84–0.91) for BCC. For Samsung 10 the AUROCs were 0.85 (95% CI: 0.79–0.90) and 0.87 (95% CI, 0.83–0.90), and for the iPhone 11 they were 0.88 (95% CI, 0.84–0.93) and 0.89 (95% CI, 0.86–0.92) for SCC and BCC, respectively. Using pre-determined diagnostic thresholds on images taken on the iPhone 6S the AIaMD achieved a sensitivity and specificity of 98% (95% CI, 88–100%) and 38% (95% CI, 33–44%) for SCC; and 94% (95% CI, 90–97%) and 28% (95 CI, 21–35%) for BCC. All 16 lesions diagnosed as melanoma in the study were correctly classified by the AIaMD.

**Discussion:**

The AIaMD has the potential to support the timely diagnosis of malignant and premalignant skin lesions.

## 1. Introduction

Non-Melanoma Skin Cancer (NMSC) is the fifth most common form of all types of cancer worldwide, with the most common NMSC types being Basal Cell Carcinoma (BCC), accounting for 75% of cases, and Squamous Cell Carcinoma (SCC), accounting for 23% of NMSC cases (1). In the UK, there are around 156,000 NMSC cases diagnosed, resulting in 920 deaths, *per annum*. The actual incidence of NMSC may be higher however, as it is known to be under-reported due to the number of multiple diagnoses per patient. Incidence rates of skin cancer have increased by over 2.5-fold (169%) since the early 1990s and are projected to rise by 14% in the UK between 2023 and 2025 (2). While NMSCs make up most of skin cancer diagnoses, melanoma has a much higher mortality rate due to high risk of metastasis, and early diagnosis is critical. When melanoma is caught early, the chances of survival are greatly improved (3).

Currently, diagnosis of NMSC is usually clinical, with subsequent histological confirmation following excision and specialist interpretation (4). To facilitate early diagnosis, alongside managing patient concern, a high proportion of ‘suspicious moles’ are referred from primary care on the two-week wait pathway, which has seen an increase from 332-thousand referrals in 2015/16 to 509-thousand referral in 2019/20 (5). However, a high proportion of these lesions are benign (6) with the main diagnoses being melanocytic naevi or seborrheic keratosis. Due to the nature of these referrals, they are awarded an inappropriate priority at the expense of more serious disorders. As a result, healthcare services are under pressure with the number of patients being referred for specialist evaluation, onward biopsies and subsequent management of suspicious skin lesions, such that a decreasing percentage of patients referred on a two-week wait pathway are seen within 14 days (5). There is a need to improve diagnostic accuracy of skin lesions earlier on in this process, in order to minimize unnecessary referrals and skin biopsies.

Deep Ensemble for the Recognition of Malignancy (DERM) is a Digital Health Technology that includes an Artificial Intelligence as a Medical Device (AIaMD) algorithm that is able to analyze dermoscopic images of a skin lesion and determine the presence of melanoma in pigmented lesions, with a similar accuracy to clinicians specialized in skin cancer detection (7). The AIaMD has been trained and tested on dermoscopic images of skin lesions with confirmed diagnoses of a range of malignant and non-malignant lesions and sub-types. This helps ensure that, for example, melanoma lesions with different clinical appearance like amelanotic melanoma (8), would be classified as melanoma. However, the AIaMD would not be expected to identify skin cancer from different image types, such as that from reflectance confocal microscopy. The AIaMD is also able to detect BCC and SCC, premalignant and selected benign lesions [such as Intraepidermal Carcinoma (IEC/SCC *in situ*), actinic keratosis, seborrheic keratosis, and benign melanocytic nevi] providing additional information to aid the clinician in differentiating skin cancers, including melanoma, from benign conditions. The AIaMD provides a high degree of accuracy in the diagnosis of NMSC using historical dermoscopic images, but clinical validation is necessary to demonstrate its utility in clinical practice. DERM is a Class IIa UKCA marked medical device and has been deployed in clinical pathways within the UK since 2020.

## 2. Materials and methods

The DERM-003 study was a prospective, multi-center, single-arm, cross-sectional, blinded study (NCT04116983), designed to demonstrate the effectiveness of the AIaMD to identify SCC and BCC. Secondary objectives included demonstrating the effectiveness of the AIaMD to identify premalignant and benign conditions, comparing the AIaMD performance to dermatologists, and demonstrating the feasibility of image capture in a clinic setting. Ethical approval for the study was granted by the Leicester South National Research Ethics committee.

Eligible participants were patients attending dermatology clinics with at least one suspicious skin lesion that was suitable for photographing. Lesions were defined as suspicious by a dermatologist, with no requirement on lesions being of a particular type or pigmentation. Patients provided written informed consent for the study. Recruitment was on a consecutive, competitive recruitment basis in 4 UK hospitals between June 2020 and February 2022. Lesions needed to be less than 15 mm in diameter, not located on an anatomical site unsuitable for photographing (genitals, hair-bearing areas, under nails) or in an area of visible scarring or tattooing, and not previously biopsied, excized or otherwise traumatized. Suitable lesions were photographed by three smartphones (iPhone 6S, iPhone 11 Apple Inc., Samsung Galaxy S10) with (dermoscopic image) or without (macroscopic image) a Dermlite DL1 Basic (DermLite LLC) lens attached, providing a 10x magnification. In addition, one dermoscopic image of healthy skin was also taken by each camera. The AIaMD assessment was not shared with the investigator, who managed the patient in accordance with standard of care. The patient had completed the protocol-defined procedures once the photographs had been taken. For each lesion included in the study, a clinical diagnosis and the clinician’s assessment of the likelihood of skin cancer, using a four-point Likert scale (unlikely, equivocal, likely, highly likely), was collected. Where a biopsy was taken, the histopathology-confirmed diagnosis was collected and categorized as melanoma, SCC, BCC, IEC, Actinic Keratosis (AK), Atypical, Benign or other. When there was histopathological uncertainty in the diagnosis, investigators reported the most likely diagnosis. ‘Other’ diagnoses were reviewed by the Chief Investigator.

Images of skin lesions were captured electronically and securely transferred to DERM for analysis by the AIaMD. All images were analyzed by DERM v3 after the completion of the study. The AIaMD generates a numeric output (continuous scale) for each of the examined classes, which reflects its confidence that the lesion is that condition. The sum of the numeric output of all classes is always 1. Threshold settings are defined for each lesion type, above which a lesion is classified as that lesion type. The AIaMD returns the most serious lesion type where the confidence score is above the threshold setting.

### 2.1. Statistical aspects

Patients and lesions that did not meet the inclusion criteria were excluded from the Intention To Treat population (ITT), as were those lesions without a final diagnosis available. Lesions with no AIaMD result available (missing dermoscopic images, and/or where these failed the DERM v3 image quality assessment) were excluded from the Per Protocol (PP) population. The primary analyses were conducted on biopsied lesions in the PP population only.

Area Under the Receiver Operator Characteristic (AUROC) curves were used to examine the association of the algorithm’s confidence scale with the histopathology-confirmed diagnosis (biopsied lesions) or clinical diagnosis (non-biopsied lesions). The co-primary outcome measures of the study were the one-against-all AUROC for both SCC and BCC. The iPhone 6S camera was used as the reference device. The study aimed to demonstrate both co-primary endpoints were above 0.9.

Assuming the true AUROC curve of the AIaMD is 0.98 and an incidence rate of 11% for SCC and 43% for BCC, a sample size of 45 SCC and 50 BCC lesions was required to demonstrate the AUROCs were superior to 0.9 at alpha = 0.05, with 90% power. A sample size of 543 patients, with an average of 1.2 lesions per patient, was expected to provide sufficient numbers of lesions diagnosed as SCC and BCC, but recruitment remained open until 45 SCC lesions had been included in the study.

Diagnostic accuracy indices (sensitivity, specificity, predictive values, false-positive rates, and false-negative rates) were calculated using decision thresholds determined prior to the image analysis, and applying the hierarchy within the AIaMD. The hierarchy means that, if the AIaMD identifies a lesion as potentially either a BCC or melanoma, it will return the classification of melanoma. Therefore, for a lesion diagnosed as SCC, an output from the AIaMD of “suspected melanoma” is considered a true positive, whereas for a lesion diagnosed as melanoma, an output from the AIaMD of “suspected SCC” is a false negative. The definition of true positive will therefore vary depending on the lesion type being assessed. The likelihood assessment scale was used to calculate a clinician AUROC that could be compared to the AIaMD.

The influence of patient and lesion variables that may affect the AIaMD’s accuracy were investigated. The following co-variates were examined: age, sex, Fitzpatrick skin type, skin cancer risk factors including past medical history of skin cancer, lesion body location, experience of reviewing clinician, lesion change, patient’s level of concern, clinician’s assessment of likelihood of skin cancer, malignancy sub-type and staging.

A *p*-value of <0.05 was regarded as statistically significant, and all tests were two-tailed. Statistical estimates of accuracy are reported with 95% Confidence Intervals (CIs). Statistical analysis was conducted using R language version 4.1.3 (The R Project for Statistical Computing).

## 3. Results

**Figure 1:**
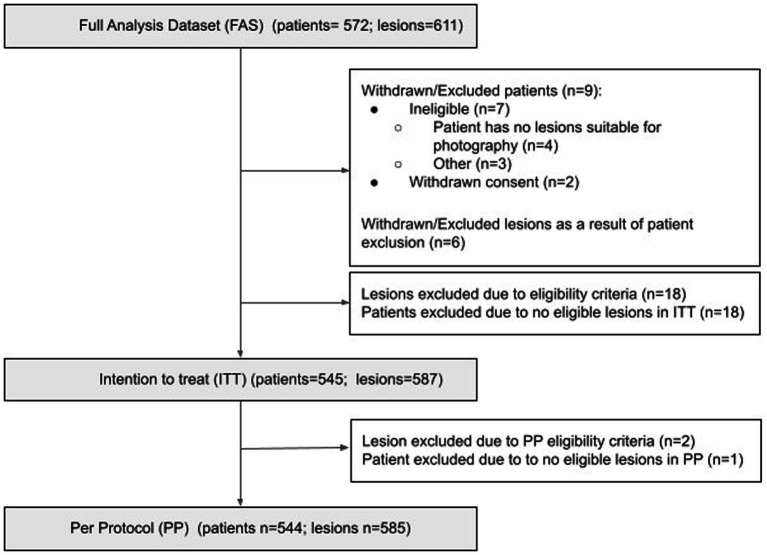
Consort diagram. Number of patients in the ITT/PP population = number of patients who have at least one lesion that fulfills the ITT/PP inclusion criteria for at least one capture device; Number of lesions in the ITT/PP datasets = number of lesions from patients included in the ITT/PP population, that fulfill the ITT/PP inclusion criteria for at least one capture device.

A total of 572 patients consented to the study, providing 611 suspicious lesions. Nine patients (6 lesions) were withdrawn / excluded from the study. Eighteen lesions were excluded from the ITT population due to failing to meet eligibility criteria, resulting in 18 patients being excluded due to no eligible lesions. Two further lesions were excluded from the PP population due to missing AIaMD results, resulting in 1 further patient being excluded from the PP population (Figure 1). Of the lesions included in the PP population, 96.7% had images available from all three combinations of hardware, 2.9% had 2 images available, and 2 lesions had just one image available. Nine images failed image quality checks.

The PP population was equally distributed between females and males, mostly White (94%) and ranged in age from 18 to 97 years (median 73). Most patients (97.8%) had Fitzpatrick skin type I-III, with over half (56.8%) the patients reporting having Fitzpatrick skin type II (Table 1). Most lesions were located on the face and scalp (46.3%), posterior chest and back (14.5%), arms (13.5%), and legs (12.3%). On average, lesions were 8.9 (±3.5 standard deviation) mm in size, ranging from 0.8 to 15 mm (Table 2).

**Table 1:** Patient demographics by analysis population.

		FA (N)	ITT (N)	PP (N)
Total		572	545	544
Sex	Female	283	273	272
	Male	286	272	272
	Missing	3	0	0
Age	Mean	68.5	68.4	68.4
	SD	17.3	17.4	17.3
	Median	73	73	73
	Minimum	18	18	18
	Maximum	97	97	97
Ethnicity	White	534	512	511
	Asian	9	8	8
	Black	3	2	2
	Mixed	1	1	1
	Other	1	1	1
	Missing/Not stated	24	21	21
Fitzpatrick skin type	I	115	113	113
	II	327	309	309
	III	112	110	110
	IV	8	8	7
	V&VI	7	5	5
	Missing	3	0	0
Past medical history	Melanoma	38	37	37
	SCC	54	51	51
	BCC	127	126	126
	Other skin cancer	6	6	6
	None	332	313	312
	Unknown	15	12	12
Family medical history	Melanoma	27	27	26
	SCC	4	4	4
	BCC	23	23	23
	Other skin cancer	30	27	27
	None	439	418	418
	Unknown	49	46	46

FA, Full Analysis; ITT, Intention-to-Treat; PP, Per Protocol; SD, Standard Deviation. Family history of skin cancer is defined as first degree family only.

**Table 2:** Lesion characteristics by analysis population.

		FA (N)	ITT (N)	PP (N)
Total		611	587	585
No. of Lesions assessed (count = number of participants)	1	532	505	504
	2	38	38	38
	3	2	2	2
Lesion size (mm)	Mean	9	8.6	8.6
	SD	4.9	3.5	3.5
	Median	8	8	8
	Minimum	0.8	0.8	0.8
	Maximum	64	20	20
Lesion location	Face and scalp	281	271	271
	Neck	21	21	21
	Anterior chest and abdomen	56	55	54
	Posterior chest and back	90	85	85
	Arms, excluding palms	80	79	79
	Palms	1	1	1
	Legs, excluding soles	80	73	72
	Soles	2	2	2
Patient level of concern	Not concerned	144	138	138
	A little concerned	307	299	299
	Very concerned	135	126	124
	Unknown	25	24	24
Experience of reviewing clinician	Foundation doctor	55	54	54
	Specialty registrar	20	19	18
	Consultant	455	440	439
	Other/GPwSI	81	74	74
	Missing	0	0	0
Lesion change	None	110	104	104
	Changed color	20	20	20
	Symptomatic	179	172	172
	Grown a bit	112	109	108
	New lesion	160	154	154
	Grown a lot	30	28	27
Clinician assessment of likelihood of skin cancer	Unlikely	224	216	215
	Equivocal	61	59	59
	Likely	211	203	202
	Highly likely	115	109	109
Biopsy taken	Lesion not referred for biopsy	167	163	162
	Further clinical review determined no biopsy needed	7	7	7
	Biopsy taken	418	398	397
	Patient refused biopsy	5	5	5
	Other	14	14	14

FA, Full Analysis; ITT, Intention-to-Treat; PP, Per Protocol; SD, Standard Deviation; GPwSI, General Practitioner with Special Interest. Number of lesions equates to number of lesion records created in the study database, the lesion count is based on clinician provided information on the number of lesions they assessed for each patient.

Forty-three lesions in the PP population were diagnosed as SCC and 176 as BCC (Table 3) by histopathology. A further 22 lesions were diagnosed as SCC or BCC by clinical diagnosis only, which were excluded from the primary analysis. These lesions did not undergo a biopsy because either the dermatologist chose to treat the lesion (*n* = 10), the patient refused biopsy (*n* = 3) or other reason (*n* = 9), including the biopsy occurred outside the study window. The PP population also included 16 lesions diagnosed as melanoma, and two lesions diagnosed as other malignancies [one Neuroendocrine, and one Spitzoid tumor of uncertain malignant potential (STUMP)] (Supplementary Table 1). Most malignancies were at an early stage.

**Table 3:** Breakdown of lesion diagnoses in the PP population.

Diagnosis	Subtype/stage	Clinical diagnosis	Histopathology
Melanoma	All	0	16
	Superficial spreading		9
	Lentigo maligna		1
	Other		1
	Not given/ambiguous		5
	*In situ*		2
	<1.0 mm		7
	1.01–2.0 mm		2
	2.01–4.0 mm		4
	>4 mm		0
	Not available		1
SCC	All	1	43
	Poorly differentiated		4
	Moderately differentiated		15
	Well differentiated		16
	Other/unknown		8
	Tis		1
	T1		38
	T2		0
	T4		3
	Not available		1
BCC	All	21	176
	Superficial		13
	Nodular		94
	Infiltrative		17
	Morphoeic		0
	Micronodular		2
	Basosquamous		1
	Other/unknown		49	
	Tis		3
	T1		141
	T2		2
	T4		0
	Not available		30
Other malignant		0	2
IEC		0	11
Actinic keratosis		40	21
Dysplastic nevus	All	2	20
	Mild atypia		9
	Moderate atypia		4
	Severe atypia		2
	Unknown severity		5
Seborrheic keratosis		59	12
Dermatofibroma		8	7
Vascular lesion		3	0
Lentigo		0	1
Benign melanocytic nevi		10	12
Other (benign)		43	75
Unknown/missing		1	1
Total lesions		188	397

SCC, Squamous Cell Carcinoma; BCC, Basal Cell Carcinoma; IEC, Intraepidermal Carcinoma.

**Figure 2:**
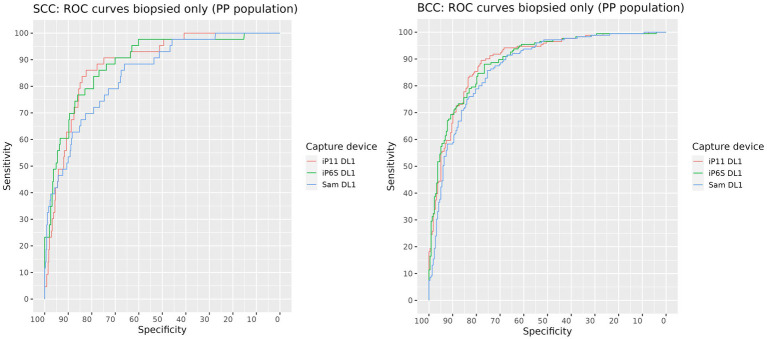
ROC curves for SCC (left) and BCC (right) produced by the AIaMD when assessing images of biopsied lesions, taken by different cameras.

**Figure 3:**
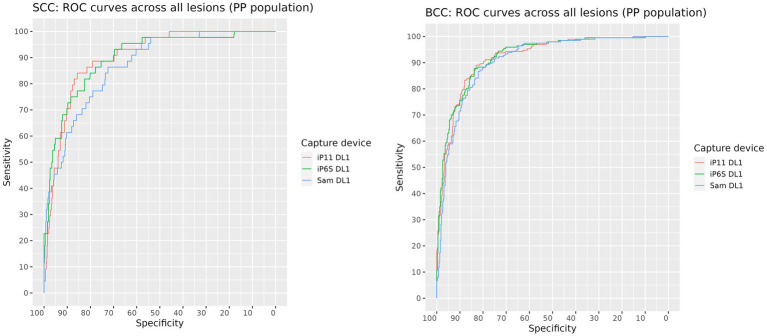
ROC curves for SCC (left) and BCC (right) produced by the AIaMD when assessing images of all lesions, taken by different cameras.

The AUROC for SCC and BCC produced on images of biopsied lesions captured on each camera were: iPhone 6S 88.5% (95% CI: 83.9–93.1%) and 89.6% (95% CI: 86.5–92.7%) respectively; iPhone 11 88.9% (95% CI: 83.8–94.0%) and 89.5% (95% CI: 86.4–92.6%) respectively; and Samsung S10 84.9% (95% CI: 79.1–90.7%) and 87.2% (95% CI: 83.8–90.7%) respectively (Figure 2 and Table 4). The AUROCs for BCC and SCC, when calculated on all lesions, were > 90% except for SCC in images captured on the Samsung 10 camera, where the AUROC was 87% (Figure 3). The AUROC for benign lesions produced by the AIaMD when assessing biopsied lesions only was between 74.9–76.8%, while the AUROC for benign lesions when all lesions were assessed, ranged between 79.8–80.9%. The AUROC for melanoma was ≥91.8% for all cameras when the AIaMD assessed both biopsied lesions and all lesions. Moderate concordance (72.9% percentage agreement) was found between the AIaMD output label using images from the two iPhones; between iPhone 6S and Samsung 10 the percentage agreement of the AIaMD output label was 60.3%, and between the iPhone 11 and Samsung 10, it was 61.7%.

**Table 4:** AUROCs produced by DERM, using images taken on each camera.

	iPhone 11 (95% CI)		iPhone 6S (95% CI)		Samsung 10 (95% CI)	
Lesions	Biopsied	All	Biopsied	All	Biopsied	All
Melanoma	91.8% (82.9–100%)	92.6% (84.3–100%)	97.5% (94.8–100%)	97.5% (94.8–100%)	94.4% (89.2–99.6%)	94.6% (89.9–99.3%)
SCC	88.5% (83.9–93.1%)	90.1% (86.1–94.0%)	88.9% (83.8–94.0%)	90.0% (85.3–94.7%)	84.9% (79.1–90.7%)	87.0% (82.1–91.9%)
BCC	89.6% (86.5–92.7%)	92.0% (89.7–94.3%)	89.5% (86.4–92.6%)	92.3% (90.1–94.6%)	87.2% (83.8–90.7%)	90.9% (88.4–93.3%)
IEC	87.7% (82.0–93.4%)	89.0% (84.2–93.8%)	81.2% (73.3–89.2%)	83.3% (76.6–90.1%)	78.2% (67.8–88.6%)	80.2% (71.1–89.3%)
AK	77.3% (66.7–87.9%)	81.1% (75.0–87.2%)	86.1% (78.5–93.7%)	82.8% (77.0–88.7%)	77.8% (68.4–87.3%)	76.4% (69.6–83.3%)
Atypical	91.5% (85.4–97.5%)	89.4% (82.7–96.2%)	93.9% (87.0–100%)	93.0% (86.1–99.9%)	80.2% (68.3–92.1%)	80.9% (70.6–91.3%)
Benign	75.2% (69.9–80.6%)	80.9% (77.3–84.5%)	76.8% (71.6–81.9%)	80.4% (76.8–83.9%)	74.9% (69.3–80.4%)	79.8% (76.1–83.5%)

AUROC, Area Under the Receiver Operator Characteristic Curve; SCC, Squamous Cell Carcinoma; BCC, Basal Cell Carcinoma; IEC, Intraepidermal Carcinoma; AK, Actinic keratosis; CI, Confidence Intervals. Because of the necessity for a dermoscopic image of the lesion to be available for assessment by DERM, the number of lesions included was different for each camera.

The AUROC for SCC and BCC produced by clinicians were 74.0% (95% CI: 66.4–81.6%) and 85.6% (95% CI: 81.8–89.3%) for biopsied lesions, and 76.9% (95% CI: 69.6–84.3%) and 90.0% (95% CI: 87.3–92.7%) for all lesions, respectively (Table 5). The AUROCs for SCC lesions were significantly lower than those produced by the AIaMD (*p* < 0.026 for each camera). The clinician AUROCs were also significantly lower than those produced by the AIaMD (*p* ≤ 0.04) for lesions diagnosed as IEC, AK and benign by histopathology. A weak to moderate level of agreement between clinical and histopathology diagnosis labels was found (percentage agreement 66.4%; Cohen’s kappa = 0.52, *p* < 0.001).

**Table 5:** AUROC of clinician assessment of likelihood of skin cancer.

Class	Biopsied lesions (95% CI)	All lesions (95% CI)
Lesions (N)	396	583
Melanoma	90.2% (80.2–100%)	90.3% (80.4–100%)
SCC	74.0% (66.4–81.6%)	76.9% (69.6–84.3%)
BCC	85.6% (81.8–89.3%)	90.0% (87.3–92.7%)
IEC	63.6% (49.8–77.4%)	63.6% (49.8–77.4%)
AK	56.9% (49.2–64.6%)	85.0% (79.2–90.8%)
Atypical	83.2% (72.3–94%)	85.1% (75.1–95%)
Benign	67.1% (62.2–72%)	82.1% (78.8–85.5%)

AUROC, Area Under the Receiver Operator Characteristic Curve; SCC, Squamous Cell Carcinoma; BCC, Basal Cell Carcinoma; IEC, Intraepidermal Carcinoma; AK, Actinic keratosis; CI, Confidence Intervals.

When pre-set threshold settings were applied, the sensitivity of the AIaMD to identify malignant lesions was above 90%, and the specificity of the AIaMD for malignant lesions was above 41.5% for each individual malignant lesion type and for all malignant lesions (Table 6). Both “other malignant” lesions were classified as malignant by the AIaMD using images from all cameras. The sensitivity and specificity of the AIaMD was more variable for other lesion types, particularly atypical lesions where the sensitivity varied between 38.1% for the Samsung and 86.4% for the iPhone 6S. In comparison, when considering the suspected diagnosis documented by the clinician at the time of their assessment, they labeled fewer melanoma and SCC lesions accurately compared to the AIaMD (melanoma sensitivity of 81.2% compared to >93% by the AIaMD, SCC sensitivity of 63.6% compared to >90%), and more BCC lesions (sensitivity of 97.5% compared to <96%). Conversely, clinicians achieved a much higher specificity for malignant lesions and were more accurate at identifying benign lesions than the AIaMD.

**Table 6:** Diagnostic performance metrics of clinicians and DERM, using images from each camera, for all lesions in the Per Protocol population.

	Device	Lesions (N)	Sensitivity (95% CI)	Specificity (95% CI)	PPV (95% CI)	NPV (95% CI)	FNR (95% CI)	FPR (95% CI)
Melanoma	Clinicians	581	81.2% (53.7–95.0%)	98.9% (97.6–99.6%)	68.4% (43.5–86.4%)	99.5% (98.3–99.9%)	18.8% (5.0–46.3%)	1.1% (0.4–2.4%)
	iPhone 6S	578	100% (74.7–100%)	69.6% (65.6–73.4%)	8.1% (4.7–13.2%)	100% (98.8–100%)	0% (0–25.3%)	30.4% (26.6–34.4%)
	iPhone 11	571	93.3% (66.0–99.7%)	73.6% (69.6–77.1%)	8.7% (5.0–14.4%)	99.8% (98.4–100%)	6.7% (0.3–34.0%)	26.4% (22.9–30.4%)
	Samsung	578	100% (75.9–100%)	65.5% (61.4–69.4%)	7.6% (4.6–12.3%)	100% (98.7–100%)	0% (0–24.1%)	34.5% (30.6–38.6%)
SCC	Clinicians	565	63.6% (47.7–77.2%)	89.1% (86–91.5%)	32.9% (23.4–44.1%)	96.7% (94.5–98.0%)	36.4% (22.8–52.3%)	10.9% (8.5–14.0%)
	iPhone 6S	563	95.4% (83.3–99.2%)	44.7% (40.4–49.1%)	12.8% (9.5–17%)	99.2% (96.6–99.9%)	4.6% (0.8–16.7%)	55.3% (50.9–59.6%)
	iPhone 11	556	93.2% (80.3–98.2%)	45.7% (41.3–50.1%)	12.8% (9.5–17.1%)	98.7% (96–99.7%)	6.8% (1.8–19.7%)	54.3% (49.9–58.7%)
	Samsung	562	90.9% (77.4–97%)	50.6% (46.2–55%)	13.5% (9.9–18.1%)	98.5% (95.9–99.5%)	9.1% (3–22.6%)	49.4% (45–53.8%)
BCC	Clinicians	521	97.5% (93.9–99.1%)	77.4% (72.4–81.8%)	72.6% (66.7–77.7%)	98% (95.2–99.3%)	2.5% (0.9–6.1%)	22.6% (18.2–27.6%)
	iPhone 6S	519	94.9% (90.6–97.4%)	41.6% (36.2–47.2%)	49.9% (44.7–55%)	93.1% (87.3–96.4%)	5.1% (2.6–9.4%)	58.4% (52.8–63.8%)
	iPhone 11	512	95.8% (91.7–98%)	45% (39.5–50.6%)	51.1% (45.8–56.4%)	94.7% (89.5–97.5%)	4.2% (2–8.3%)	55% (49.4–60.5%)
	Samsung	518	94.4% (89.9–97%)	54.5% (48.9–60%)	55.6% (50.1–61%)	94.1% (89.4–96.9%)	5.6% (3.0–10.1%)	45.5% (40–51.1%)
Malignant	Clinicians	583	93.8% (90–96.3%)	77.4% (72.4–81.8%)	77% (71.9–81.4%)	94.3% (90.6–96.7%)	5.8% (3.4–9.5%)	22.6% (18.2–27.6%)
	iPhone 6S	580	95.7% (92.3–97.7%)	41.6% (36.2–47.2%)	56.8% (52–61.5%)	92.4% (86.5–96%)	4.3% (2.3–7.7%)	58.4% (52.8–63.8%)
	iPhone 11	573	96.0% (92.6–98%)	45% (39.5–50.6%)	58% (53.1–62.7%)	93.5% (88.1–96.7%)	4% (2–7.4%)	55% (49.4–60.5%)
	Samsung	580	94.9% (91.3–97.2%)	54.5% (48.9–60%)	62.4% (57.4–67.2%)	93.1% (88.3–96.1%)	5.1% (2.8–8.7%)	45.5% (40–51.1%)
IEC	Clinicians	323	90.9% (57.1–99.5%)	78.8% (73.8–83.2%)	13.2% (6.8–23.3%)	99.6% (97.4–100%)	9.1% (0.5–42.9%)	21.1% (16.8–26.2%)
	iPhone 6S	322	100% (67.9–100%)	43.1% (37.5–48.8%)	5.9% (3.1–10.5%)	100% (96.5–100%)	0% (0–32.1%)	56.9% (51.2–62.5%)
	iPhone 11	320	100% (67.9–100%)	46.6% (41–52.3%)	6.2% (3.3–11.2%)	100% (96.8–100%)	0% (0–32.1%)	53.4% (47.7–59%)
	Samsung	323	90.9% (57.1–99.5%)	56.1% (50.4–61.6%)	6.8% (3.5–12.5%)	99.4% (96.4–100%)	9.1% (0.5–42.9%)	43.9% (38.4–49.6%)
AK	Clinicians	312	96.7% (87.6–99.4%)	79.3% (73.6–84%)	53.1% (43.5–62.6%)	99% (96.1–99.8%)	3.3% (0.6–12.4%)	20.7% (16–26.4%)
	iPhone 6S	311	85.0% (72.9–92.5%)	43.4% (37.2–49.8%)	26.4% (20.5–33.3%)	92.4% (85.6–96.2%)	15% (7.5–27.1%)	56.6% (50.2–62.8%)
	iPhone 11	309	84.8% (72.5–92.4%)	47.2% (40.9–53.6%)	27.5% (21.3–34.7%)	92.9% (86.6–96.5%)	15.2% (7.6–27.5%)	52.8% (46.4–59.1%)
	Samsung	312	83.6% (71.5–91.4%)	51.4% (45–57.7%)	29.5% (22.9–37%)	92.8% (86.8–96.3%)	16.4% (8.6–28.5%)	48.6% (42.3–55%)
Atypical	Clinicians	251	76.2% (52.5–90.9%)	73.9% (67.6–79.4%)	21% (12.9–32.2%)	97.1% (93.1–98.9%)	23.8% (9.1–47.5%)	26.1% (20.6–32.4%)
	iPhone 6S	251	86.4% (64.0–96.4%)	39.3% (33.0–46.0%)	12% (7.6–18.4%)	96.8% (90.2–99.2%)	13.6% (3.6–36.0%)	60.7% (54.0–67.0%)
	iPhone 11	250	59.1% (36.7–78.5%)	43.9% (37.4–50.6%)	9.2% (5.2–15.6%)	91.7% (84.5–95.9%)	40.9% (21.5–63.3%)	56.1% (49.4–62.6%)
	Samsung	251	38.1% (19.0–61.3%)	48.3% (41.7–54.9%)	6.3% (3.0–12.4%)	89.5% (82.4–94.1%)	61.9% (38.7–81.0%)	51.7% (45.1–58.3%)
Premalignant	Clinicians	323	91.4% (83.3–95.9%)	73.9% (67.6–79.4%)	58.6% (50.1–66.6%)	95.5% (91.0–97.9%)	8.6% (4.1–16.7%)	26.1% (20.6–32.4%)
	iPhone 6S	322	87.1% (78.2–92.9%)	39.3% (33.0–46.0%)	36.8% (30.5–43.6%)	88.2% (80.0–93.5%)	12.9% (7.1–21.8%)	60.7% (54.0–67.0%)
	iPhone 11	320	80.4% (70.6–87.7%)	43.9% (37.4–50.6%)	36.6% (30.1–43.7%)	84.8% (76.7–90.5%)	19.6% (12.3–29.4%)	56.1% (49.4–62.6%)
	Samsung	323	75.3% (65.0–83.4%)	48.3% (41.7–54.9%)	37% (30.2–44.4%)	82.8% (75.1–88.6%)	24.7% (16.6–35.0%)	51.7% (45.1–58.3%)
Benign	Clinicians	581	73.9% (67.6–79.4%)	93.7% (90.5–95.9%)	88.5% (83.0–92.5%)	84.6% (80.5–87.9%)	26.1% (20.6–32.4%)	6.3% (4.1–9.5%)
	iPhone 6S	578	39.3% (33.0–46.0%)	94.3% (91.1–96.4%)	81.8% (73.1–88.3%)	70.3% (65.9–74.4%)	60.7% (54.0–67.0%)	5.7% (3.6–8.9%)
	iPhone 11	571	43.9% (37.4–50.6%)	93.3% (90.0–95.6%)	81.3% (73.1–87.5%)	71.4% (67.0–75.5%)	56.1% (49.4–62.6%)	6.7% (4.4–10.0%)
	Samsung	578	48.3% (41.7–54.9%)	91.4% (87.8–94.0%)	78.7% (70.9–85.0%)	72.8% (68.3–76.8%)	51.7% (45.1–58.3%)	8.6% (6–12.2%)

SCC, Squamous Cell Carcinoma; BCC, Basal Cell Carcinoma; IEC, Intraepidermal Carcinoma; AK, Actinic Keratosis; CI, Confidence Intervals Rate; PPV, Positive Predictive Value; NPV, Negative Predictive Value; FPR, False Positive Rate; FNR, False Negative.

Univariate analyses and multiple logistic regression analyses were performed on the FA population, filtered for those images with a final diagnosis available, to identify patient and lesion characteristics that might have influenced the accuracy of the AIaMD results and clinical diagnosis. Age above 60 was associated with a non-significant reduction in the accuracy of both dermatologists and the AIaMD to identify malignant lesions in images from the iPhones (Odds Ratio (OR) = 0.37–0.88, *p* > 0.16) and minor improvement in images from the Samsung 10 (OR = 1.07–1.18, *p* > 0.7). The impact only reached significance (*p* = 0.034) for the AIaMD with images from the iPhone 11, in patients aged 74–82. No significant impact was seen for either the AIaMD assessment or clinicians to accurately identify malignant lesions due to the Fitzpatrick skin type, however no cancers were detected in patients with Fitzpatrick skin types V and VI. Indeed, the only factor associated with a significant improvement on the accuracy of dermatologists to identify malignant lesions was a likely or high likelihood of skin cancer (OR > 7, *p* < 0.018), and on the AIaMD was a high level of patient concern (OR = 1.95, *p* = 0.008).

## 4. Discussion

The DERM-003 study is the first prospective, powered, clinical validation study that specifically evaluates the ability of the AIaMD to identify NMSC. Previously, the performance of the AIaMD to identify melanoma was evaluated (7), though this was on an earlier version of the software which focused solely on the identification of melanoma. DERM v3 is designed to identify SCC and BCC, alongside melanoma, as well as a range of premalignant, atypical and benign lesions often mistaken for skin cancer. The study recruited patients in dermatology clinics across the UK, such that the population reflects the aging, primarily Caucasian, population seen in these clinics. Although patients with Fitzpatrick Skin types V and VI were recruited, no skin cancers were diagnosed in these patients. Indeed, only 2.2% of the study population had Fitzpatrick skin type IV-VI, limiting the generalizability of these results for patients with darker skin tones. However, this reflects the trend seen in other clinical studies, and in the real world, where few patients with Fitzpatrick skin types IV-VI are seen in dermatology clinics with suspicious skin lesions (7, 9) and as such the study population can be seen as representative of the population that DERM would be used on. Robust performance evaluation of technologies, such as DERM, in patients with darker skin types may only be possible through post-market surveillance analyses, where more patients with these skin types can be evaluated (10). Similarly, the study included lesions across a good distribution of body locations, including those with higher sun exposure (head, neck upper body) and lower limbs, where lesions can look different, and a range of skin cancer sub-types and stages that are seen in dermatology clinics. The study also included two “other malignant” lesions, which were diagnosed as STUMP and neuroendocrine, and a range of benign lesions.

When the study was designed, the calculations used to determine the success criteria and sample size were based on *in silica* performance data, which provided an assumption that the true AUROC for both SCC & BCC was 98%. The clinical performance of AI-based devices has frequently been shown to be lower than that of laboratory-based data (11–13), and as such an expectation that the true AUROC achieved by the AIaMD on fresh clinical data would be comparable to laboratory results was perhaps unrealistic. Although the study failed to meet either of the co-primary endpoints, the AUROCs achieved by the AIaMD for SCC and BCC were still high and at least comparable to dermatologists. Indeed, the AUROCs of the clinical diagnosis for SCC and BCC lesions do not achieve a 90% AUROC either, indicating that even between clinician and histology there is a huge amount of diagnostic variability. This may be a reflection of clinical practice, where uncertainty of diagnosis drives a conservative view and decision to biopsy. Reassuringly, the AUROC produced by the AIaMD for melanoma was higher than that previously reported (7), demonstrating an improved performance of the AIaMD over the earlier version of the algorithm.

It should be noted that for non-biopsied lesions, the clinical diagnosis was used as the ground truth against which both the AIaMD and clinical diagnosis were compared. Clinical diagnosis therefore will appear more accurate in an all-lesion population, compared to a biopsy-only population, for those lesions where a high proportion do not have a histopathology diagnosis, specifically BCC, AK, and benign lesions. Despite this, the AUROCs achieved by the AIaMD for non-malignant lesions are comparable to those achieved by dermatologists in an all-lesion population, and indeed are notably higher than dermatologists in a biopsy only population.

The study assessed the performance of the AIaMD on images captured by three smartphone cameras available in the UK market at the time of the study. They were chosen to demonstrate performance of the AIaMD across different physical hardware devices (camera specification), operating systems, and price points and included a reference combination (iPhone 6S/DL1) which Skin Analytics has used in a previous study (7). Across the three cameras, the AUROCs for melanoma, SCC and BCC were very similar, indicating a good generalizability of the algorithm across the image capture hardware used. Although a greater variability across the cameras is seen for non-malignant lesions, the AUROCs achieved by the AIaMD from all cameras are still high.

The thresholds used to determine the sensitivity and specificity of the AIaMD were defined to be suitable for use in a secondary care setting at the beginning of the study. The sensitivity achieved by the AIaMD for melanoma, SCC and all malignant lesions were higher than achieved by clinical diagnosis alone, though clinicians referred these lesions for biopsy, so their management decision ensured a sensitivity of 100%. Even for BCC, sensitivity achieved by the AIaMD was around 95% using images from all cameras, and the sensitivity and specificity of the AIaMD to identify premalignant and atypical lesions are at a level that are clinically useful. Additionally, the specificity and NPV values for malignant lesions indicate that the AIaMD could aid the appropriate management of benign lesions. The threshold settings used in live deployments of the AIaMD are different than used in this study, and the sensitivity across all malignant lesions achieved in the real world have been demonstrated to be even higher (10), demonstrating the value in optimizing the settings within the AIaMD for the population it is being used to assess. The sensitivities achieved by the AIaMD for non-malignant lesions are more variable across the cameras than seen for malignant lesions, specifically atypical and benign lesions. Similarly, there was only a moderate concordance between the outputs produced by the AIaMD when analyzing images captured by the different image capture hardware. This may be due to variances in the hardware and post-processing software, or a factor of the threshold settings used by the AIaMD to assign the output label. If the confidence scores produced by the AIaMD on images of the same lesion taken on two different cameras were similar, but fell either side of the threshold set, the AIaMD output label from each image could be different. Since the AUROCs for these lesions were similar, this suggests that the thresholds applied could be optimized for the image capture hardware being used, to achieve the best sensitivity.

The multivariate analysis identified a different impact of patient factors on the accuracy of malignant lesion detection by the AIaMD compared with previously reported analyses (7). This may reflect a change in how the AIaMD works between the two versions assessed. However, since the impact of patient factors on the accuracy of dermatologists is also different, it may be more a reflection that the previous study focused on melanoma detection, whereas this analysis considered all malignant lesions included in the study population. Further analyses are needed to understand whether these translate into a clinically relevant reduction in sensitivity and/or specificity of the AIaMD in different patient groups.

The main limitation to the DERM-003 study is the clinical setting in which it was conducted, and therefore the population studied. The study was conducted in UK secondary care dermatology clinics in order to include sufficient numbers of SCC and BCC lesions in the study population, and to easily capture the histopathology confirmed diagnosis of biopsied lesions and a dermatologist’s clinical assessment of the lesion. This means the study population was made up of patients and lesions that dermatologists determined were suitable for inclusion in the study, which may not be representative of all patients and lesions that would be assessed by DERM. For example, lesions that were clearly benign may have been excluded by a study dermatologist, but on which a less experienced clinician may use DERM to support their patient management decision. That said, the study recruited a broader spectrum of lesions in the study population compared to a previous study (7), where the study population was limited to patients with a pigmented lesion that was due for biopsy. The results of this study are therefore more generalizable to the population of patients seen in secondary care in the UK. Indeed, data from ongoing post-market surveillance monitoring indicates that DERM can be deployed safely as an adjuvant tool in live clinical services accessible to patients with eligible skin lesions (i.e., excluding those under nails, on genitalia or on hairy areas of skin), from a broad range of age groups and most representative skin types with suspicious skin lesions, with sensitivity and specificity in-line with target thresholds and performance demonstrated in clinical studies (10).

Finally, the reliance on clinical diagnosis as the ground truth for non-biopsied lesions not only artificially increases the performance metrics for the dermatologists, as discussed above, but potentially impacts the apparent performance of the AIaMD on non-biopsied lesions. The clinical diagnosis of skin cancer by clinicians is based on the subjective interpretation of morphological features and as such variability in the clinical diagnoses given by dermatologists is known to exist (14). The reliance on one dermatologist to provide the clinical diagnosis used as the ground truth for non-biopsied lesions introduces a potential bias to the results for both the AIaMD and dermatologists. The use of a panel of dermatologists to provide a consensus diagnosis would have provided a greater confidence in the clinical diagnosis ground truth, and provided an independent diagnosis against which to compare the investigating dermatologist.

In conclusion, even though the study failed to meet its co-primary endpoints, the results from the DERM-003 study showed that the AIaMD can detect NMSC and premalignant lesions with a similar level of accuracy as dermatologists, and that taking the images was a quick and well tolerated process. DERM could provide dermatologist level assessment of suspicious skin lesions earlier in the patient pathway, potentially enabling the earlier diagnosis of malignant lesions and improvement of differentiation between harmless and potentially harmful lesions by non-specialists.

## Data availability statement

The raw data supporting the conclusions of this article will be made available by the authors, without undue reservation.

## Ethics statement

The studies involving humans were approved by Leicester South National Research Ethics Committee. The studies were conducted in accordance with the local legislation and institutional requirements. The participants provided their written informed consent to participate in this study.

## Author contributions

HM: Conceptualization, Funding acquisition, Investigation, Methodology, Project administration, Writing – original draft. CM: Data curation, Investigation, Writing – review & editing. SA: Data curation, Investigation, Writing – original draft. CD: Data curation, Investigation, Writing – review & editing. MV: Data curation, Formal analysis, Investigation, Methodology, Writing – review & editing. CK: Conceptualization, Formal analysis, Methodology, Writing – review & editing. JG: Methodology, Software, Writing – review & editing. DM: Investigation, Resources, Supervision, Writing – review & editing. IP: Conceptualization, Investigation, Supervision, Writing – review & editing. SA: Writing - reviewing and editing.
