# Supplementary material for: Effectiveness of an image analyzing AI-based Digital Health Technology to identify Non-Melanoma Skin Cancer and other skin lesions: results of the DERM-003 study
Source: Front Med (Lausanne). 2023 Oct 6;10:1288521. doi: 10.3389/fmed.2023.1288521 (PMC10587678; doi:10.3389/fmed.2023.1288521)
Supplement: Supplementary file 1 [file Table_1.DOCX]

Supplementary Table 1: Breakdown of “Other” diagnoses, as determine by histopathology or clinical diagnosis.

| Lesion type | Diagnosis source | Diagnosis | Count (N) |
| --- | --- | --- | --- |
| Other “Malignant” | Histopathology | Neuroendocrine | 1 |
|  |  | Spitzoid tumor of uncertain malignant potential (STUMP) | 1 |
| Other “Benign” | Histopathology | Keratoacanthoma | 9 |
|  |  | Intradermal melanocytic naevus | 7 |
|  |  | Viral wart | 7 |
|  |  | Compound naevus | 6 |
|  |  | Non-specific inflammation | 6 |
|  |  | Unknown / ambiguous | 6 |
|  |  | Haemangioma | 5 |
|  |  | Chondrodermatitis nodularis helicis | 4 |
|  |  | Scar tissue | 4 |
|  |  | Lichenoid keratosis | 3 |
|  |  | Lichenoid dermatitis | 2 |
|  |  | Sebaceoma | 2 |
|  |  | Benign squamoproliferative lesion | 1 |
|  |  | Blue naevus | 1 |
|  |  | Cyst | 1 |
|  |  | Eccrine Poroma | 1 |
|  |  | Fibrous papule | 1 |
|  |  | Freckle | 1 |
|  |  | Hyperplasia | 1 |
|  |  | Hypertrophic lichen planus | 1 |
|  |  | Lichenoid planus | 1 |
|  |  | Molluscum contagiosum | 1 |
|  |  | Ruptured suppurative folliculitis | 1 |
|  |  | Sebaceous hyperplasia | 1 |
|  |  | Trichilemmoma | 1 |
|  |  | Ulcer | 1 |
|  | Clinical Diagnosis | Viral wart | 7 |
|  |  | Sebhorreic wart | 6 |
|  |  | Bowenoid AK | 4 |
|  |  | Haemangioma | 3 |
|  |  | Lichenoid keratosis | 3 |
|  |  | Cyst | 2 |
|  |  | Venous lake | 2 |
|  |  | Seborrheic and actinic keratosis | 2 |
|  |  | Benign mole | 1 |
|  |  | Blue nevus | 1 |
|  |  | Cherry angioma | 1 |
|  |  | Chondrodermatitis nodularis helicis | 1 |
|  |  | Dysplastic naevus/?MM | 1 |
|  |  | ?SebK ? BCC | 1 |
|  |  | Excoriations | 1 |
|  |  | Inflamed Seborrheic Keratosis | 1 |
|  |  | Intra dermal lesion | 1 |
|  |  | Keratotic nodule | 1 |
|  |  | SCC KA type | 1 |
|  |  | Sebaceous hyperplasia | 1 |
|  |  | Solar lentigo | 1 |
|  |  | Spider naevus | 1 |
